# Magnetoliposomes Containing Calcium Ferrite Nanoparticles for Applications in Breast Cancer Therapy

**DOI:** 10.3390/pharmaceutics11090477

**Published:** 2019-09-14

**Authors:** Daniela S. M. Pereira, Beatriz D. Cardoso, Ana Rita O. Rodrigues, Carlos O. Amorim, Vítor S. Amaral, Bernardo G. Almeida, Maria-João R. P. Queiroz, Olga Martinho, Fátima Baltazar, Ricardo C. Calhelha, Isabel C. F. R. Ferreira, Paulo J. G. Coutinho, Elisabete M. S. Castanheira

**Affiliations:** 1 Centre of Physics (CFUM), University of Minho, Campus de Gualtar, 4710-057 Braga, Portugal; daniela.s.pereira30@gmail.com (D.S.M.P.); beatrizdiascardoso94@gmail.com (B.D.C.); ritarodrigues@fisica.uminho.pt (A.R.O.R.); bernardo@fisica.uminho.pt (B.G.A.); pcoutinho@fisica.uminho.pt (P.J.G.C.); 2 Physics Department and CICECO, University of Aveiro, Campus de Santiago, 3810-193 Aveiro, Portugal; amorim5@ua.pt (C.O.A.); vamaral@ua.pt (V.S.A.); 3 Centre of Chemistry, University of Minho, Campus de Gualtar, 4710-057 Braga, Portugal; mjrpq@quimica.uminho.pt; 4 Life and Health Sciences Research Institute (ICVS), School of Medicine, University of Minho, Campus de Gualtar, 4710-057 Braga, Portugal; olgamartinho@med.uminho.pt (O.M.); fbaltazar@med.uminho.pt (F.B.); 5 ICVS/3B’s-PT Government Associate Laboratory, 4710-057 Braga/4806-909 Guimarães, Portugal; 6 Mountain Research Centre, Polytechnic Institute of Bragança, Campus de Santa Apolónia, 5300-253 Bragança, Portugal; calhelha@ipb.pt (R.C.C.); iferreira@ipb.pt (I.C.F.R.F.)

**Keywords:** calcium ferrite nanoparticles, magnetoliposomes, new antitumor drugs, thienopyridine derivatives, breast cancer therapy

## Abstract

Magnetoliposomes containing calcium ferrite (CaFe_2_O_4_) nanoparticles were developed and characterized for the first time. CaFe_2_O_4_ nanoparticles were covered by a lipid bilayer or entrapped in liposomes forming, respectively, solid or aqueous magnetoliposomes as nanocarriers for new antitumor drugs. The magnetic nanoparticles were characterized by UV/Visible absorption, XRD, HR-TEM, and SQUID, exhibiting sizes of 5.2 ± 1.2 nm (from TEM) and a superparamagnetic behavior. The magnetoliposomes were characterized by DLS and TEM. The incorporation of two new potential antitumor drugs (thienopyridine derivatives) specifically active against breast cancer in these nanosystems was investigated by fluorescence emission and anisotropy. Aqueous magnetoliposomes, with hydrodynamic diameters around 130 nm, and solid magnetoliposomes with sizes of ca. 170 nm, interact with biomembranes by fusion and are able to transport the antitumor drugs with generally high encapsulation efficiencies (≥70%). These fully biocompatible drug-loaded magnetoliposomes can be promising as therapeutic agents in future applications of combined breast cancer therapy.

## 1. Introduction

Cancer still represents a leading cause of morbidity and mortality worldwide, although new treatments have improved the prognosis of cancer patients over the past years [1]. According to the WHO, cancer was responsible for 8.8 million deaths in 2015, representing the second leading cause of death [2]. Therefore, oncology is the first therapeutic area covered by nanomedicine [3]. Considering the possibility of targeted chemotherapy, simultaneous hyperthermia and individualized therapies, the so-called “magic bullets” are one of the most promising therapeutic approaches. These “magic bullets” can selectively target cancerous tissues, as well as remotely release antitumor drugs at optimal dosage. Numerous formulations based on different materials have been investigated to design the “magic bullet” formulation with the best properties, including, for example, liposomes, micelles, hydrogels, polymers, and magnetic nanoparticles [4]. The use of magnetic nanoparticles has been widely investigated for their ability to target specific sites of interest, produce local heat, and act as contrast agents [5]. However, toxicity issues have been raised, pointing to the need of fully biocompatible nanomaterials [6]. Therefore, increasing attention has been given to the synthesis of magnetic nanoparticles without transition metals on their constituents. Ions such as Ca^2+^ and Mg^2+^ have been proposed to substitute transition metals in the crystalline structure of ferrite nanoparticles, as they may promote a higher biocompatibility [7]. Calcium ferrite nanoparticles have shown to be biocompatible at concentrations below 250 mg/mL, in in vitro cytotoxicity tests on T-cell lines, exhibiting enhanced cell viability when compared to other ferrites [8]. The high heating capacity of calcium ferrite and calcium/magnesium ferrite nanoparticles also make them suitable for magnetic hyperthermia [9,10].

Magneto-sensitive liposomes result from the incorporation of magnetic nanoparticles into liposomes (aqueous magnetoliposomes) or from the covering of a nanoparticles cluster with a lipid bilayer (solid or dry magnetoliposomes, SMLs). These magnetoliposomes can overcome individual problems of liposomes and magnetic nanoparticles and, at the same time, gather the outstanding properties of each in a single nanosystem [11]. Recently, magnetoliposomes based on several types of superparamagnetic ferrite nanoparticles have been developed, namely iron oxide [12,13,14,15,16,17], manganese ferrite [18,19], nickel ferrite [20], and magnesium ferrite nanoparticles [21].

**Figure 1:**
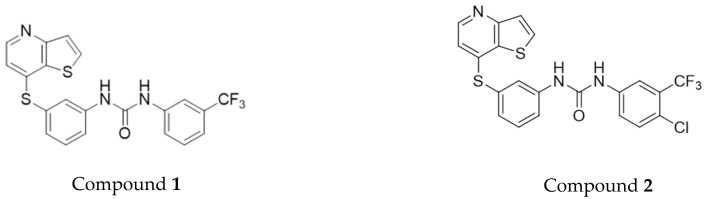
Structure of the 1-aryl-3-[3-(thieno[3,2-*b*]pyridin-7-ylthio)phenyl]urea derivatives.

Pursuing both high biocompatibility and heating capability, together with the ability to transport antitumor drugs, in this work magnetoliposomes based on calcium ferrite nanoparticles were developed. These systems were tested as nanocarriers for new potential antitumor drugs, diarylurea derivatives of thienopyridine (Figure 1). Thienopyridine-based compounds have been described as promising antitumoral agents and receptor tyrosine kinase inhibitors [22,23]. Moreover, a high antiproliferative activity on human breast cancer cell lines, MCF-7 (hormone dependent breast adenocarcinoma) and MDA-MB-231 (hormone independent, triple-negative breast cancer), was described for these compounds [24], with very low growth inhibitory concentrations for 50% of cells, GI_50_ (Table 1). Therefore, drug-loaded magnetoliposomes containing biocompatible calcium ferrite nanoparticles and the antitumor compounds **1** or **2** can be promising nanosystems for breast cancer therapy.

## 2. Materials and Methods

All the solutions were prepared using spectroscopic grade solvents and ultrapure water of Milli-Q grade (MilliporeSigma, St. Louis, MO, USA).

### 2.1. Preparation of Calcium Ferrite Nanoparticles

Calcium ferrite (CaFe_2_O_4_) nanoparticles were synthesized in 2 mL aqueous solution, by the co-precipitation method, as previously described by Hirazawa et al. [9]. First, a basic aqueous solution of 50% NaOH was heated to 90 °C. Then, a mixture of solutions, 0.5 M calcium acetate dihydrate and 1.0 M FeCl_3_·6H_2_O solution was added, drop by drop, to the previously warmed basic solution under magnetic stirring. After two hours at 90 °C, calcium ferrite nanoparticles were formed. For purification, the obtained sample was washed several times by centrifugation (14,000 *g*) and magnetic decantation. Then, the samples were dried overnight at 60 °C. A calcination step at 600 °C was carried out for 30 min [9]. At this temperature, the formation of brownmillerite (Ca_2_Fe_2_O_5_) is avoided [9].

### 2.2. Preparation of Magnetoliposomes

For magnetoliposomes preparation, the lipids l-α-phosphatidylcholine from egg yolk (Egg-PC), and 1,2-dipalmitoyl-*sn*-glycero-3-phosphocholine (DPPC), from Sigma-Aldrich (St. Louis, MO, USA), were used in a final concentration of 1 mM. The ethanol injection method was employed to obtain aqueous magnetoliposomes (AMLs) [25]. Accordingly, a 20 mM lipid solution in ethanol was injected, under vigorous vortexing, to an aqueous dispersion of magnetic nanoparticles (with 4 mg/mL concentration). After encapsulation, the ferrofluid was washed with water and purified by magnetic decantation to remove all the non-encapsulated nanoparticles. Considering the strong difference in maximum magnetization of magnetic nanoparticles and aqueous magnetoliposomes (as previously reported [26,27]), due to the diamagnetic contribution of water, the aqueous magnetoliposomes are not attracted to the magnet. Therefore, only non-encapsulated magnetic nanoparticles are separated by this technique, keeping the magnetoliposomes in the supernatant phase. The lipid phase (in the supernatant) remains unchanged upon decantation, the initial and final lipid concentrations being the same.

For the preparation of solid magnetoliposomes, a method previously developed was used [19,20]. First, 10 μL of a solution of the synthesized magnetic nanoparticles (0.02 mg/mL) were ultrasonicated for one minute at 189 W, and 3 mL of chloroform were added to the solution. Then, immediately after vigorous agitation, 150 μL of a 20 mM methanolic solution of the lipid DPPC (1,2-dipalmitoyl-*sn*-glycero-3-phosphocholine) were injected under vortexing, to form the first lipid layer of the SMLs. To remove the lipid that was not attached to the nanoparticles surface, the particles were washed twice by magnetic decantation with ultrapure water. The lipid bilayer was completed by a new injection of 150 μL of 20 mM lipid methanolic solution, under vortexing, in 3 mL of aqueous dispersion of the particles with the first lipid layer. The solid magnetoliposomes obtained were then washed and purified with ultrapure water by magnetic decantation.

The antitumor thieno[3,2-*b*]pyridine derivatives **1** or **2** were incorporated into aqueous magnetoliposomes by simultaneous injection of compound and lipid (co-injection method), in a final compound concentration of 5 μM. Neat liposomes (without magnetic nanoparticles) with encapsulated compounds were also prepared in the same manner. In solid magnetoliposomes, the compound was incorporated by injection of an ethanolic solution (0.2 mM) immediately before the formation of the second lipid layer.

### 2.3. Preparation of Giant Unilamellar Vesicles (GUVs)

GUVs of soybean lecithin (l-α-phosphatidylcholine from soybean), from Sigma-Aldrich (St. Louis, MO, USA), were obtained by the thin film hydration method, as described by Tamba et al. [28,29]. A lipid film of 100 μL of soybean lecithin solution (1 mM) was obtained by solvent evaporation under an ultrapure nitrogen stream, and 40 μL of water were added, followed by incubation at 45 °C for 30 min. Then, 3 mL of glucose aqueous solution (0.1 M) were added and the resulting solution was again incubated at 37 °C for 2 h. After incubation, the GUVs suspension was centrifuged at 14,000 *g* for 30 min at 20 °C, to remove multilamellar vesicles and lipid aggregates.

### 2.4. Spectroscopic Measurements

#### 2.4.1. General Methods

Absorption spectra were performed in a Shimadzu UV-3600 Plus UV-vis-NIR (Shimadzu Corporation, Kyoto, Japan) spectrophotometer. Fluorescence measurements were recorded using a Horiba Fluorolog 3 spectrofluorimeter (HORIBA Jobin Yvon IBH Ltd., Glasgow, UK), equipped with double monochromators in both excitation and emission, Glan-Thompson polarizers and a temperature controlled cuvette holder. Fluorescence spectra were corrected for the instrumental response of the system.

#### 2.4.2. FRET Measurements

**Figure 2:**
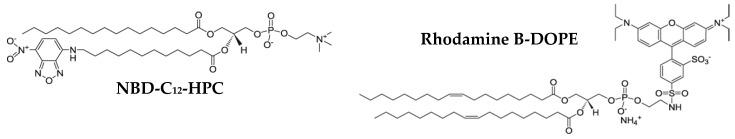
Structure of the fluorescent labeled lipids used in Förster resonance energy transfer (FRET) assays.

Förster resonance energy transfer (FRET) assays were used to confirm the formation of the lipid bilayer in the solid magnetoliposomes containing CaFe_2_O_4_ nanoparticles. For that purpose, the nitrobenzoxazole labeled lipid NBD-C_12_-HPC, 1-palmitoyl-2-{12-[(7-nitro-2-1,3-benzoxadiazol- 4-yl)-amino]hexanoyl}-*sn*-glycero-3-phosphocholine (from Avanti Polar Lipids, Alabaster, AL, USA) was included in the first lipid layer, while the rhodamine B labeled lipid Rhodamine B-DOPE (1,2-dioleoyl-*sn*-glycero-3-phosphoethanol-amine-*N*-lissamine rhodamine B sulfonyl (ammonium salt)) (from Avanti Polar Lipids, Alabaster, AL, USA) was included the second lipid layer (structures of the labeled lipids in Figure 2).

FRET efficiency, Φ_RET_, defined as the proportion of donor molecules that have transferred their excess energy to the acceptor molecules, was calculated through donor emission quenching, by taking the ratio of the donor integrated fluorescence intensities in the presence of acceptor (F_DA_) and in the absence of acceptor (F_D_) (Equation (1)) [30],

(1) $$\Phi_{\mathrm{RET}}=1-\frac{F_{\mathrm{DA}}}{F_{D}}$$

The distance between the donor and acceptor molecules was determined through the FRET efficiency (Equation (2)),

(2) r=R0[1−ΦRETΦRET]16,

where *R*_0_ is the Förster radius (critical distance), that can be obtained by the spectral overlap, J(λ), between the donor emission and the acceptor absorption, according to Equations (3) and (4) (with *R*_0_ in Å, λ in nm, ε_A_(λ) in M^−1^·cm^−1^) [30],

(3) R0=0.2108[k2ΦD0n−4J(λ)]16,

(4) $$J\left(\lambda \right)=\int_{0}^{\infty }I_{D}(\lambda )\varepsilon_{A}\left(\lambda \right)\lambda^{4}d\lambda ,$$

where k2=23 is the orientational factor assuming random orientation of the dyes, *n* is the refraction index of the medium, *I*_D_(λ) is the fluorescence spectrum of the donor normalized so that $\int_{0}^{\infty }I_{D}\left(\lambda \right)d\lambda =1$, and ε_A_(λ) is the molar absorption coefficient of the acceptor. $\Phi_{D}^{0}$, the fluorescence quantum yield of the donor in the absence of energy transfer, was determined by the standard method (Equation (5)) [31,32],

(5) $$\Phi_{D}^{0}=\frac{A_{r}F_{D}n_{D}^{2}}{A_{D}F_{r}n_{r}^{2}}\Phi_{r},$$

where *A* is the absorbance at the excitation wavelength, *F* the integrated emission area and *n* the refraction index of the solvents used. Subscripts refer to the reference (r) or donor (D). The absorbance at the excitation wavelength was always lower than 0.1 to avoid the inner filter effects. The NBD-C_12_-HPC molecule intercalated in lipid membranes was used as reference, Φ_r_ = 0.32 at 25 °C, as reported by Invitrogen [33].

**Figure 3:**
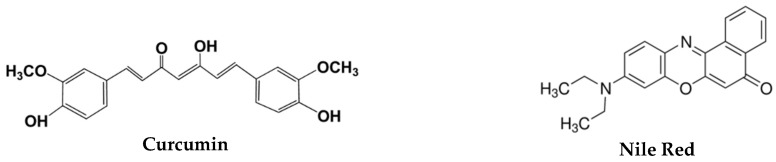
Structures of the dyes used in FRET assays for interaction with giant unilamellar vesicles (GUVs).

The hydrophobic dyes curcumin (energy donor) and Nile Red (energy acceptor) (Figure 3) were also incorporated in magnetoliposomes for monitoring the interaction of magnetoliposomes with giant unilamellar vesicles (GUVs) by FRET.

#### 2.4.3. Fluorescence Anisotropy Measurements

The steady-state fluorescence anisotropy, *r*, is calculated by

(6) $$r=\frac{I_{\mathrm{VV}}-GI_{\mathrm{VH}}}{I_{\mathrm{VV}}+2GI_{\mathrm{VH}}},$$

where *I*_VV_ and *I*_VH_ are the intensities of the emission spectra obtained with vertical and horizontal polarization, respectively (for vertically polarized excitation light), and $G=I_{\mathrm{HV}}/I_{\mathrm{HH}}$ is the instrument correction factor, where *I*_HV_ and *I*_HH_ are the emission intensities obtained with vertical and horizontal polarization (for horizontally polarized excitation light).

#### 2.4.4. Drug Encapsulation Efficiency

The encapsulation efficiency, *EE*(%), of the potential antitumor drugs in magnetoliposomes, was determined through fluorescence emission measurements. For that, drug loaded magnetoliposomes were subjected to centrifugation at 11,000 rpm for 60 min using Amicon^®^ Ultra centrifugal filter units 100 kDa (Merck Millipore, Darmstadt, Germany). Then, the filtrate (containing the non-encapsulated drug) was pipetted out, the water was evaporated and the same amount of ethanol was added. After vigorous agitation, its fluorescence was measured, allowing to determine the drug concentration using a calibration curve (fluorescence intensity vs. concentration) previously obtained in the same solvent. Three independent measurements were performed for each system and standard deviations (SD) were calculated. The encapsulation efficiency was determined using Equation (7),

(7) $$EE\left(\%\right)=\frac{\left(total\;amount-amount\;of\;non\;encapsulated\;compound\right)}{total\;amount}\times 100$$

### 2.5. Structural Characterization

#### 2.5.1. Electron Microscopy (TEM and SEM)

HR-TEM images of magnetic nanoparticles were recorded using a Transmission Electron Microscope JEOL JEM 2010F (JEOL Ltd., Tokyo, Japan) operating at 200 kV coupled to an Electron Dispersive Spectroscopic analyzer (EDS) at C.A.C.T.I (Centro de Apoio Científico e Tecnolóxico á Investigación), Vigo, Spain. SEM images of solid magnetoliposomes were recorded using a Scanning Electron Microscope FEI—Nova 200 NanoSEM (FEI Technologies, Inc., Hillsboro, OR, USA), operating in transmission mode (STEM). For SMLs, a negative staining was employed, using a 2% aqueous solution of ammonium molybdate tetrahydrate. 20 μL of sample and 20 μL of staining solution were mixed and a drop of the mixture was placed onto a Formvar grid (Agar Scientific Ltd, Essex, UK), held by tweezers. After 20 s, almost all the solution was removed with filter paper and left dry. TEM images were processed using ImageJ software (National Institutes of Health (NIH), Bethesda, MD, USA) [34]. The area of each particle allowed an estimation of the particle diameter. The resulting histogram was fitted to a Gaussian distribution.

#### 2.5.2. X-Ray Diffraction (XRD)

X-ray diffraction (XRD) analyses were performed using a conventional Philips PW 1710 (Royal Philips, Amsterdam, The Netherlands) diffractometer, operating with Cu Kα radiation, in a Bragg-Brentano configuration.

#### 2.5.3. Dynamic Light Scattering (DLS)

The mean diameter and size distribution (polydispersity index) of aqueous and solid magnetoliposomes (1 mM lipid concentration) were measured using a dynamic light scattering (DLS) equipment NANO ZS Malvern Zetasizer (Malvern Panalytical Ltd., Malvern, UK) at 25 °C, using a He-Ne laser of λ = 632.8 nm and a detector angle of 173°. Five independent measurements were performed for each sample.

### 2.6. Magnetic Measurements

Magnetization measurements were done in a MPMS3 SQUID magnetometer MPMS5XL (Quantum Design Inc., San Diego, CA, USA). The hysteresis cycles (magnetization versus magnetic field) of the samples were measured in the convenient field range for each sample. The measurement method was by DC extraction or VSM oscillation at a frequency of 14 Hz. A specific magnetic field correction for the trapped flux in the superconducting coil was made achieving an accuracy of residual less than 2 Oe.

### 2.7. Nanoparticles Encapsulation Efficiency

The nanoparticles encapsulation efficiency in aqueous magnetoliposomes (AMLs) was estimated from the spectrophotometric determination of iron (III) content, through the formation of a phenylfluorone complex sensitized with Triton X-100 [35]. To obtain iron (III) from the magnetoliposomes, the latter were digested by heating at 500 °C overnight, to remove all the biological components. Then, 1 mL of concentrated nitric acid (from Sigma-Aldrich) was added and the sample was heated to 80 °C for 2 h. The temperature was then raised to 150 °C for 48 h, and the pH was increased until 5.5 by successive cycles of addition and evaporation of ultrapure water (Milli-Q grade). Finally, 1 mL of water was added to the digested sample and, after ultrasonication for one hour, the iron (III) content of magnetoliposomes was released.

For the spectrophotometric measurements, the standard addition method was employed. 100 μL of the digested sample and 2 × 10^−5^ M, 3 × 10^−5^ M, 4 × 10^−5^ M, 5 × 10^−5^ M, or 6 × 10^−5^ M of iron (III) stock solution were added to 1.6 × 10^−4^ M phenylfluorone and 4 × 10^−3^ M Triton X-100 solutions. The pH was then adjusted to 9 using 0.05 M borax buffer [35]. A calibration curve for the determination of iron (III) concentration was previously obtained. Three independent measurements were performed and standard deviations (SD) were calculated. The encapsulation efficiency, *EE*(%), of calcium ferrite nanoparticles (NPs) in AMLs was determined by equation:

(8) $$EE\left(\%\right)=\frac{\left(total\;amount\;of\;NPs-amount\;of\;nonencapsulated\;NPs\right)}{total\;amount\;of\;NPs}\times 100$$

### 2.8. Studies in Cell Lines

Compound **1** and compound **2** were tested in several cell lines, using different compound concentrations (0.03 to 125 µM). In vitro cytotoxicity evaluation was assessed for four human tumor cell lines, namely NCI-H460 (non-small cell lung cancer), HeLa (cervical carcinoma), and HepG2 (hepatic cancer). The cell line PLP2 (non-tumor cells) was used to evaluate the toxicity to healthy tissues. The cells were routinely maintained as adherent cell cultures in RPMI-1640 medium containing 10% heat-inactivated FBS, at 37 °C, in a humidified air incubator containing 5% CO_2_. Each cell line was plated at an appropriate density (1.0 × 10^4^ cells per well) in 96-well plates and allowed to attach for 24 h. The cells were then treated for 48 h with the different solutions. Following this incubation period, the adherent cells were fixed by adding cold 10% TCA (100 μL) and incubated for 60 min at 4 °C. Plates were then washed with deionized water and dried. A sulforhodamine B (SRB) solution (0.1% in 1% acetic acid, 100 μL) was then added to each plate-well and incubated for 30 min at room temperature. Unbound SRB was removed by washing with 1% acetic acid. The plates were air-dried and the bound SRB was solubilized with 10 mM Tris-HCl buffer (200 μL, pH = 7.4). The absorbance was then measured at 540 nm in a microplate reader [36,37]. The results were expressed in GI_50_ values (concentration that inhibited 50% of net cell growth).

Drug-loaded aqueous and solid magnetoliposomes were preliminary tested against breast cancer cell lines, namely MCF-7 and MDA-MB-231.

## 3. Results and Discussion

### 3.1. Properties of the Antitumor Compounds

#### 3.1.1. Studies in Cell Lines

Studies of the growth inhibitory activity in human tumor cell lines were performed to get a deeper understanding of the antitumor capabilities of compounds **1** and **2**. The assays were carried out for NCI-H460 (non-small cell lung cancer), HeLa (cervical carcinoma) and HepG2 (hepatocellular carcinoma). Assays using the non-tumor cell line PLP2 (porcine liver primary cells) were also performed to evaluate compound toxicity to healthy tissues (Table 2).

The results on Table 2 clearly show that both compounds are non-toxic for normal (non-tumor) cells (PLP2), as the GI_50_ values are generally much higher than in human tumor cell lines. Besides, the compounds show a notable specificity for breast cancer, exhibiting very low inhibitory concentrations in MCF-7 breast adenocarcinoma cells (Table 1) and also in the highly aggressive, invasive and poorly differentiated triple-negative breast cancer (MDA-MB-231 cell line) [24]. Compound **2** is slightly less active in breast cancer cell lines (Table 1), but more active in the other tumor cell lines, being also less toxic to non-tumor cells (Table 2). Therefore, the in vitro studies of cell inhibitory activity point to a potential efficacy of these thienopyridine derivatives in oncological therapies focusing breast cancer.

#### 3.1.2. Fluorescence Properties

**Figure 4:**
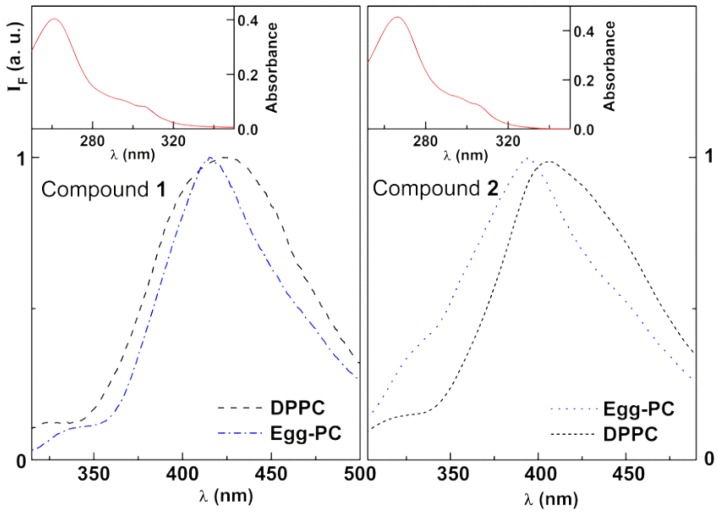
Fluorescence spectra (λ_exc_ = 270 nm) of compound **1** (left) and compound **2** (right) in liposomes of 1,2-dipalmitoyl-*sn*-glycero-3-phosphocholine (DPPC) and l-α-phosphatidylcholine from egg yolk (Egg-PC), with compound concentration 5 × 10^−6^ M (T = 25 °C). Insets: Examples of absorption spectra (compound concentration: 1 × 10^−5^ M).

Both compounds **1** and **2** exhibit fluorescence emission in several solvents but not in water, which is a common behavior to other thienopyridine derivatives [38]. However, these compounds display significant fluorescence when entrapped in liposomes, pointing to their location mainly in the lipid bilayer [38]. Figure 4 shows the fluorescence spectra of antitumor compounds **1** and **2** in neat liposomes, as well as examples of absorption spectra.

The emission spectra of both compounds show some sensitivity to the environment felt by the compound in liposomes, especially compound **2**. In DPPC liposomes, which are in the gel phase at room temperature, the thienopyridines may be located near the lipid polar head groups, feeling a more hydrated environment than in Egg-PC liposomes, which are in the fluid phase (with a deeper penetration in the bilayer of the latter). This behavior allows the use of fluorescence emission to investigate compounds location in magnetoliposomes and to determine the encapsulation efficiency in these nanocarriers.

### 3.2. Nanoparticles Characterization

As referred, the interest in calcium ferrite nanoparticles is the substitution of transition metals (Ni, Mn, Co…) in ferrite nanoparticles, due to the toxicity of these elements. No doubt, iron oxide nanoparticles have been the most widely used magnetic nanoparticles in biomedical applications and are considered biocompatible. Compound **1** was even preliminary tested in magnetoliposomes containing magnetite nanoparticles, in a previous work [39]. However, it has been reported that these nanoparticles induce the production of reactive oxygen species (ROS) in mammalian cells [40], causing severe DNA and protein damage and inflammatory responses, which may be a safety concern [40,41,42]. Therefore, the development of magnetoliposomes containing other biocompatible magnetic nanoparticles that may avoid these adverse effects is advantageous. In this respect, CaFe_2_O_4_ is a very attracting magnetic material because of its thermal and chemical stability, as well as the proven high biocompatibility [8]. Considering all these aspects, in this work, calcium ferrite nanoparticles were chosen for the preparation of magnetoliposomes, with the aim of a future application in cancer therapy.

#### 3.2.1. Absorption Spectra

**Figure 5:**
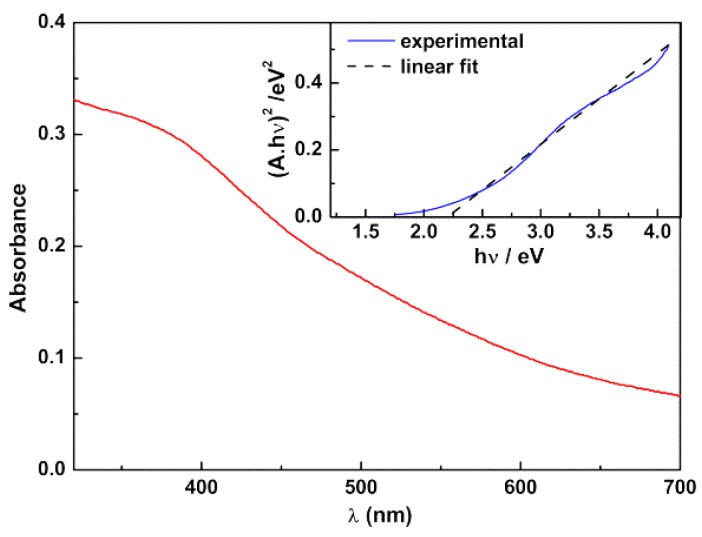
UV/visible absorption spectra of an aqueous dispersion of CaFe_2_O_4_ magnetic nanoparticles. Inset: Tauc plot.

Figure 5 displays the UV/Visible absorption spectrum of the synthesized calcium ferrite nanoparticles, exhibiting a wide wavelength range of absorption. Using a Tauc plot (Equation (9)),

(9) $${\left(\alpha h\nu \right)}^{n}\propto \left(h\nu -E_{g}\right),$$

where α is the absorption coefficient (proportional to the absorbance), *n* is an exponent that depends on the nature of the transition (being *n* = 2 for a direct semiconductor and *n* = 1/2 for an indirect one) and E_g_ is the optical band gap [43], a linear relation was obtained for *n* = 2 (direct semiconductor). A band gap of 2.19 eV was estimated from the intercept of inset of Figure 5, in agreement with the value of 1.90 eV reported by Kim et al. [44].

For the objectives of this work, the important feature is the wide absorption range of CaFe_2_O_4_ nanoparticles, which may quench drugs fluorescence emission in drug-loaded magnetoliposomes, as previously described [19,21].

#### 3.2.2. X-Ray Diffraction (XRD) Measurements

**Figure 6:**
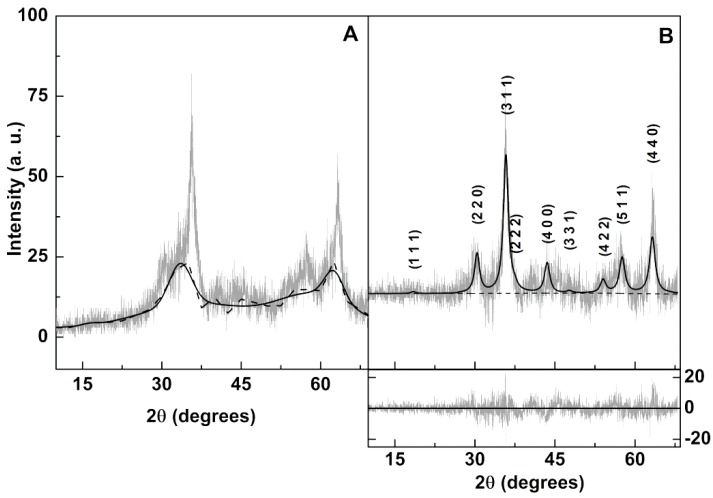
XRD diffractogram of calcium ferrite nanoparticles. (**A**) Original; (**B**) Background subtracted.

The XRD diffractogram of the prepared calcium ferrite nanoparticles shows low intensity peaks with large width characteristic of small crystalline nanoparticles (Figure 6A). The baseline is composed of broad bands indicating the presence of amorphous content. Using FullProf software suite [45], the diffractogram was fitted to a cubic spinel phase (space group Fd$\bar{3}$m, adapted from magnetite CIF 9000926), with a degree of inversion *i* = 0.85, as reported by Lal et al. [46] and using, as background, a set of 22 equally spaced points with adjustable intensities joined by linear regression lines. This yielded a very good fit, with a R_F_ value of 2.90 (Table 3). The resulting background (dashed line in Figure 6A) was then fitted to a minimal set of Gaussian functions and subtracted from the experimental XRD data. The resulting diffractogram was then analyzed using a much reduced set of 3 background points (Figure 6B). A reasonable value of R_F_ = 4.2 was obtained, confirming the synthesis of a pure crystalline phase of calcium ferrite nanoparticles, since all their characteristic peaks, marked by their indices, are observed (Figure 6B). Using the Debye-Scherrer method, as implemented by FullProf [45], the resulting average size was 5.4 nm. The lattice parameter is in accordance with values already reported for CaFe_2_O_4_ spinel structure, which are between 8.33 Å and 8.37 Å using a sol-gel combustion method [46,47], and 8.37 Å using a coprecipitation protocol [9].

#### 3.2.3. Transmission Electron Microscopy (TEM)

**Figure 7:**
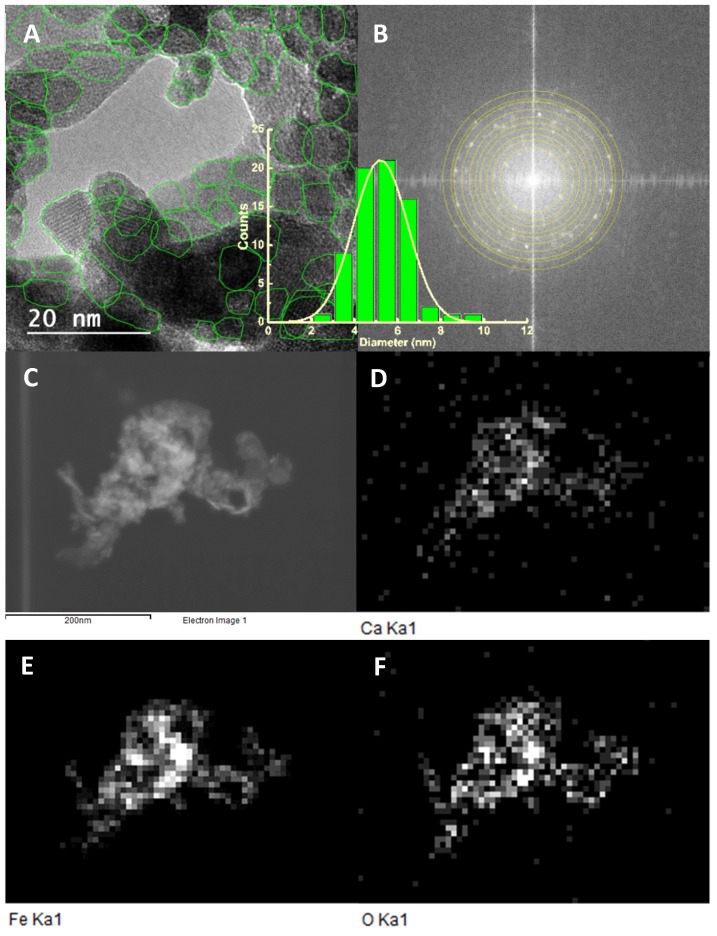
TEM images of the synthesized CaFe_2_O_4_ nanoparticles. (**A**) Selection of particles (Scale bar: 20 nm). Inset: Particles size histogram of image (**A**); (**B**) Fast Fourier transform (FFT) and diffraction rings; (**D**–**F**): EDX images of TEM image (**C**), using Ca (**D**), Fe (**E**), and O (**F**) X-ray emission lines (Scale bar is the same of image (**C**): 200 nm).

TEM images (Figure 7A) of the CaFe_2_O_4_ prepared nanoparticles reveal cuboid/spherical nanoparticles with line patterns that arise from electron beam diffraction at crystal planes. The limits of these patterns helped on the identification of individual particles, as shown by the green lines in Figure 7A. The shown histogram results from the area of 71 nanoparticles and is well described by a Gaussian distribution of 5.2 ± 1.2 nm. This is in excellent agreement with the XRD results. Figure 7B is the fast Fourier transform (FFT) of Figure 7A, using ImageJ image processing software. The bright spots allowed identifying a set of *d*-spacing values that, using a cubic fcc crystal structure (equation 10), are compatible with a lattice constant *a* = 8.37 Å and Miller indices (*h k l*) = (1 1 0), (1 1 1), (2 0 0), (1 2 0), (2 1 1), (2 2 0), (2 2 1), (2 2 2), (3 2 0), and (3 2 1).

(10) $$d_{hkl}=\frac{a}{\sqrt{h^{2}+k^{2}+l^{2}}}$$

These results are again in agreement with the previous XRD analysis of the obtained CaFe_2_O_4_ nanoparticles. A further confirmation of the identity of the nanoparticles observed in TEM images was achieved from EDX measurements (Figure 7D–F). It is clearly observed that most of the particles in Figure 7C have simultaneously Ca, Fe, and O atoms, thus being compatible with the expected calcium ferrite phase.

#### 3.2.4. Magnetic Properties

**Figure 8:**
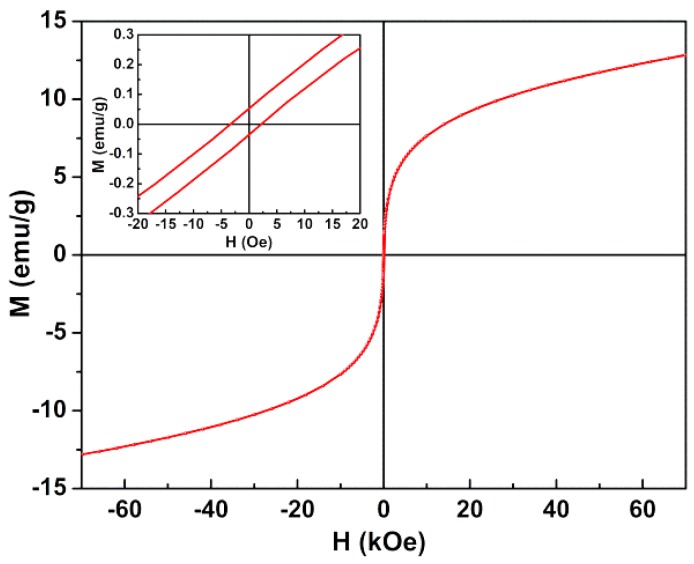
Magnetization hysteresis loop of calcium ferrite nanoparticles measured at room temperature (T = 300 K). Inset: Enlargement of the loop in the low field region.

The magnetic properties of CaFe_2_O_4_ nanoparticles were characterized by their magnetic hysteresis loop, obtained using a SQUID magnetometer, where the relation between the induced magnetic moment (*M*) and the applied magnetic field (*H*) is evidenced. The *M-H* curve at room temperature is plotted in Figure 8. From the hysteresis loop, a slight coercivity (*H*_c_) of 3.41 Oe and a low remanent magnetization (*M*_r_) of 0.06 emu/g were determined, pointing to a superparamagnetic behavior. Superparamagnetism can be related to the magnetic squareness value of the hysteresis cycle, which is the ratio between the remanescent magnetizations and the saturation magnetization (*M*_s_). If below 0.1, this ratio indicates that more than 90% of the magnetization is lost upon the removal of the applied magnetic field [48,49]. Here, the obtained magnetic squareness value of 0.005 (using the maximum magnetization of 12.81 emu/g at *H* = 70 kOe) clearly indicates that the synthesized CaFe_2_O_4_ nanoparticles are superparamagnetic at room temperature. Saturation in magnetization was not achieved at the maximum applied field of 70 kOe, which can be due to non collinear canted magnetic structure (typical of small nanoparticles with high surface-to-volume ratio).

As used in the analysis of XRD results, the obtained CaFe_2_O_4_ nanoparticles have a strongly inverted spinel structure, with an inversion degree of 0.85. Therefore, its lattice can be written as (Ca_0.15_ Fe_0.85_↑) [Ca_0.85_ Fe_1.15_↓]O_4_, where parentheses depict the cations at the tetrahedral sites and square brackets the amount of cations at octahedral ones. Lal et al. [46] have computed an expected magnetic moment per unit formula of 1.78 μ_B_ for CaFe_2_O_4_ nanoparticles with the same lattice structure. Here, a value of 0.49 μ_B_ is obtained through the relation η_B_ = (*M*_W_ × *M*_s_)/5585 (where η_B_ is the magnetic moment in Bohr units and *M*_W_ is the molecular weight) [49,50]. The difference between this estimation and the computed value of magnetic moment [46] can be attributed to the spin canting phenomenon typical of this type of ferrites [49,50,51] and to the amorphous content of the prepared nanoparticles (as evidenced from the XRD data).

The maximum magnetization of calcium ferrite nanoparticles is slightly lower than the one previously obtained for magnesium ferrite nanoparticles also prepared by coprecipitation [21], however keeping the superparamagnetic character. Despite the low maximum magnetization, these nanoparticles guarantee enhanced biocompatibility [8] and superparamagnetic characteristics, avoiding the undesirable oxidation and production of reactive oxygen species (ROS) in mammalian cells by iron oxide nanoparticles that cause DNA and protein damage and inflammatory responses [40,41,52].

### 3.3. Development of Magnetoliposomes

The obtained magnetic nanoparticles were either entrapped in liposomes (aqueous magnetoliposomes, AMLs) or covered by a lipid bilayer, forming solid magnetoliposomes (SMLs). As previously reported, solid magnetoliposomes are only suitable as carriers for hydrophobic drugs, but exhibit magnetic properties similar to those of the neat nanoparticles. On the other hand, aqueous magnetoliposomes can be used as nanocarriers for both hydrophilic and hydrophobic drugs, but their magnetic properties are poorer, due to the diamagnetic contribution of water [26,27].

#### 3.3.1. Magnetoliposomes Characterization

The calcium ferrite nanoparticles were entrapped in liposomes composed either by the phospholipid Egg-PC or DPPC. The hydrodynamic diameter and polydispersity (PDI) of these aqueous magnetoliposomes were determined by DLS (Table 4).

The nanoparticles encapsulation efficiency in aqueous Egg-PC liposomes was calculated from the spectrophotometric determination of iron (III) [35]. The encapsulation efficiency, EE(%), obtained from three independent assays, was EE(%) ± SD(%) = 70.(4) ± 9. This percentage is slightly higher than the one reported for magnetite nanoparticles using the same encapsulation method [39].

Considering solid magnetoliposomes, the phospholipid DPPC was chosen, due to its melting transition temperature (41 °C [53]) is near mild hyperthermia temperatures [54]. At this transition temperature, the bilayer liquid-crystalline phase is attained, with an increase in membrane permeability, enhancing drug release into cell membranes. Therefore, a local temperature rise promoted by the magnetic nanoparticles under an alternating magnetic field (AMF) can trigger drug release.

The values of hydrodynamic diameter determined by DLS are similar to those previously reported [21,39,55] for magnetoliposomes of the same lipids (and containing different nanoparticles), evidencing that small magnetic nanoparticles does not influence the magnetoliposomes size. The polydispersity index (PDI) is generally low and point to systems that are monodisperse in size. The diameter of magnetoliposomes must be small to guarantee an enhanced permeability and retention (EPR) effect of loaded drugs. The successful extravasation into tumors has been shown to occur for nanocarriers with sizes below 200 nm [56], which is the case of the nanosystems developed here.

**Figure 9:**
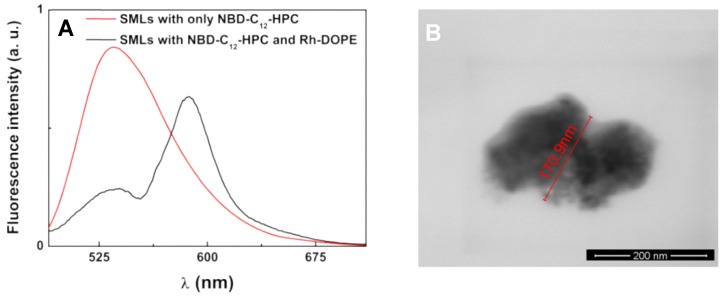
(**A**) Fluorescence spectra (λ_exc_ = 470 nm, no rhodamine excitation) of solid magnetoliposomes (SMLs) labelled with only NBD-C_12_-HPC and labeled with both NBD-C_12_-HPC and rhodamine B-DOPE; (**B**) SEM image (scale bar: 200 nm) of an aggregate of two SMLs containing CaFe_2_O_4_ nanoparticles (obtained with a negative staining).

Considering solid magnetoliposomes (SMLs), calcium ferrite nanoparticles are being used for the first time to synthesize this type of magnetoliposomes. It is, therefore, important to prove that a lipid bilayer is formed around a cluster of these magnetic nanoparticles. This can be done using FRET assays, where the labeled lipid NBD-C12-HPC (NBD as the energy donor, Figure 2) was included in the first lipid layer of SMLs and the lipid Rhodamine B-DOPE (rhodamine B as the acceptor, Figure 2) was included in the second lipid layer. The emission of SMLs containing only the NBD-labeled lipid and SMLs containing both donor and acceptor labeled lipids were measured (Figure 9A), exciting only the donor NBD. The fluorescence spectrum of SMLs with only the donor exhibits the NBD emission band (λmax = 535 nm), while the spectra of SMLs containing both donor and acceptor reveals a strong decrease in the NDB emission and the notable appearance of rhodamine B band, evidencing the occurrence of energy transfer from the excited NBD moiety to Rhodamine B. A FRET efficiency of 0.723 was estimated (using equations 2–5), corresponding to a donor-acceptor distance of 3.8 nm. These distance values prove the formation of the double lipid layer in SMLs, taking into account the usual cell membrane thickness, varying from 7 to 9 nm [57]. SEM images of these SMLs (Figure 9B) show roughly spherical structures containing nanoparticles aggregates, with sizes slightly around 170 nm, in accordance with those determined by DLS. 

#### 3.3.2. Interaction with Model Membranes

**Figure 10:**
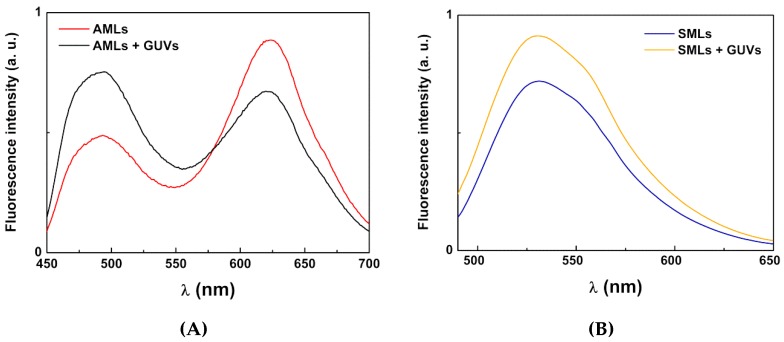
(**A**). Fluorescence spectra (λ_exc_ = 440 nm) of aqueous magnetoliposomes (AMLs) of CaFe_2_O_4_ nanoparticles containing both curcumin (2 × 10^−6^ M) and Nile Red (2 × 10^−6^ M), before and after interaction with GUVs. (**B**) Fluorescence spectra (λ_exc_ = 470 nm) of SMLs of CaFe_2_O_4_ nanoparticles containing NBD-C_12_-HPC (3 × 10^−6^ M) before and after interaction with GUVs.

For drug delivery purposes, it is also important that magnetoliposomes have some fusogenic capability with the cell membranes. This was assessed by investigating the non-specific interaction with giant unilamellar vesicles (GUVs), used as membrane models. This investigation was carried out using FRET (Förster resonance energy transfer) assays, where the aqueous magnetoliposomes (AMLs) containing two hydrophobic dyes, curcumin (acting as the energy donor, Figure 3) and Nile Red (as the energy acceptor, Figure 3), were prepared. Curcumin is a highly hydrophobic fluorescent compound [58], being located in the lipid bilayer of liposomes [21]. On the other hand, Nile Red has been widely used as lipid probe [59,60,61], the pair curcumin/Nile Red being a very favorable donor–acceptor pair in FRET assays [21]. Exciting only the donor curcumin, a strong fluorescence band due to Nile Red emission is observed (with maximum wavelength around 630 nm), resulting from energy transfer from curcumin to Nile Red (Figure 10A). After interaction with GUVs, the donor fluorescence (λ_max_ ~ 500 nm) increases and the acceptor emission band decreases, evidencing the diminution of FRET process efficiency and, consequently, proving the membrane fusion between AMLs and GUVs.

For SMLs, as previously described [19,20,21], FRET process is usually not observed due to the strong quenching of both donor and acceptor emissions by the cluster of magnetic nanoparticles. Instead, the lipid bilayer of SMLs was labeled with only a fluorescent lipid, NBD-C_12_-HPC (Figure 2). Upon interaction with GUVs, a significant rise in NBD fluorescence is observed (unquenching effect), proving the increase in the distance between the nanoparticles and the fluorescent moiety (NBD, λ_max_ = 535 nm), and thus membrane fusion occurs (Figure 10B).

### 3.4. Drug-Loaded Magnetoliposomes

**Figure 11:**
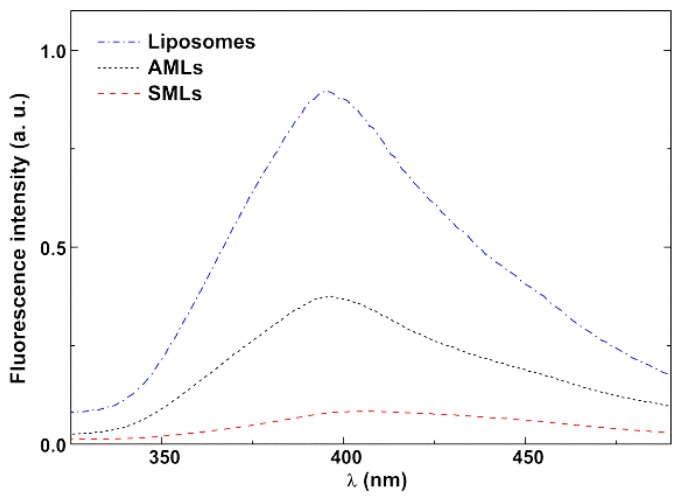
Fluorescence spectra of antitumor compound **2** in liposomes, AMLs and SMLs.

The antitumor compounds **1** and **2** were incorporated in both aqueous and solid magnetoliposomes. The two compounds exhibit fluorescence in these nanocarriers, indicating that they must be located in the lipid bilayer (as they are non-emissive in aqueous media). An emission quenching by the nanoparticles is observed for these thienopyridine derivatives, more significant in SMLs, where the cluster of nanoparticles is closer to the antitumor compound (comparing to the case of AMLs) (Figure 11). A similar behavior was previously observed for other antitumor drugs [19,21] and for compound **1** in magnetoliposomes containing iron oxide nanoparticles [39].

Fluorescence anisotropy measurements of both compounds (Table 5) were determined in liposomes (without the magnetic nanoparticles), in AMLs and in SMLs. The values in glycerol are presented for comparison, as in this highly viscous medium (η = 993.4 cP at room temperature [62]), compound molecules rotate slowly and anisotropy value approaches the fundamental anisotropy [30]. From the anisotropy results, one can observe that the values in liposomes are similar to the ones in AMLs and SMLs, pointing to the compound location in the lipid membrane in all the nanocarriers. This is not surprising, as these compounds are hydrophobic molecules. Moreover, a decrease in anisotropy is observed for DPPC at 55 °C, where the lipid bilayer is in the liquid-crystalline phase. This indicates that compounds are experiencing a higher degree of rotation in this case, with a consequent decrease in fluorescence anisotropy. Also, in phosphatidylcholine from egg yolk (Egg-PC), which is a phosphatidylcholine mixture with some unsaturated lipid chains (main components are 16:0 PC, 18:0 PC, and 18:1 PC), the anisotropy values are lower than in DPPC (16:0 PC) at room temperature, signifying larger freedom for the antitumor drugs in Egg-PC.

The drug encapsulation efficiencies were determined for both antitumor compounds in AMLs and SMLs (Table 6). The encapsulation efficiency is higher for aqueous magnetoliposomes, compound **2** being the one exhibiting greater percentages. Nevertheless, in solid magnetoliposomes, the encapsulation efficiencies are still very reasonable. Generally, the high *EE%* obtained point to a promising future utility of the drug-loaded nanocarriers containing biocompatible calcium ferrite nanoparticles in cancer therapeutics, specifically for breast cancer therapy.

**Figure 12:**
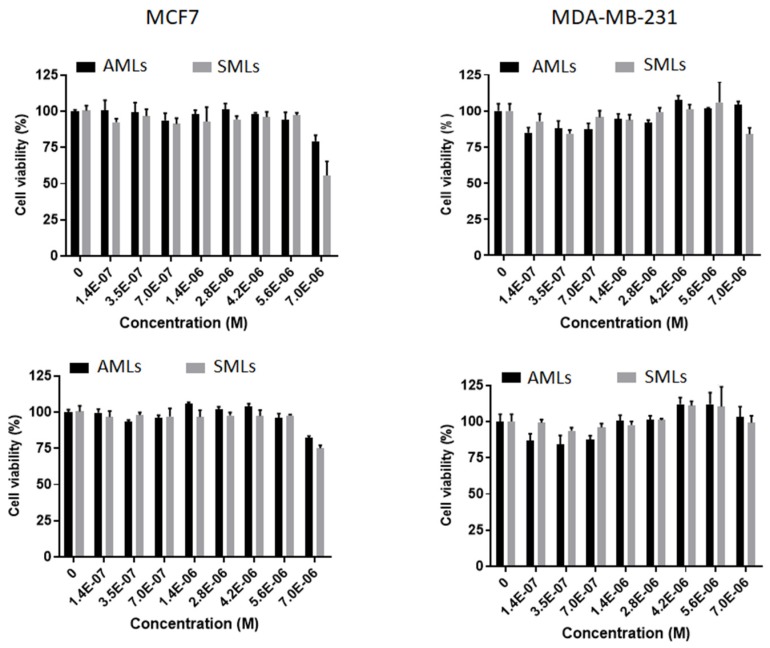
Viability of breast cancer cells MCF-7 and MDA-MB-231 in presence of drug-loaded aqueous (AMLs) and solid (SMLs) magnetoliposomes for compound **1** (above) and compound **2** (below). GI_50_ values of free drugs in breast cancer cells: MCF-7—Compound **1**: (1.2 ± 0.2) × 10^−6^ M, Compound **2**: (1.6 ± 0.3) × 10^−6^ M; MDA-MB-231—Compound **1**: (5.0 ± 0.7) × 10^−6^ M, Compound **2**: (7.0 ± 0.8) × 10^−6^ M.

Preliminary assays using drug-loaded magnetoliposomes (AMLs and SMLs) in breast cancer cell lines, MCF-7 and MDA-MB-231, were performed (Figure 12). Free (non-encapsulated) drugs were already tested in previous publications [23,24], using the same human breast cancer cell lines, with the determination of GI_50_ values [24] (values indicated in Table 1).

Figure 12 shows a generally low inhibitory activity of cells for all formulations, indicating a lack of toxicity of the drug-loaded magnetoliposomes in the absence of an alternating magnetic field (AMF). The most significant activity was observed for SMLs loaded with compound **1** in MCF-7 cell line, yet, no cytotoxicity was observed, but only a reduced cell viability. Typically, encapsulated drugs present lower bioavailability when compared to free drugs and, therefore, less cytotoxicity is observed for encapsulated drugs. In both solid and aqueous magnetoliposomes, compounds **1** and **2** remain encapsulated for more than two days (in the conditions used in cell culture) and no release is observed to the surrounding medium, which was expected considering the hydrophobic nature of the compounds. So, these assays prove that drug-loaded magnetoliposomes have practically absence of toxicity, which makes them advantageous systems for triggered drug release by application of an AMF to promote local heating, that increases liposomes membrane fluidity. Therefore, in future work, the drug-loaded magnetoliposomes (without the application of AMF) will act as a control. The results obtained here are promising for future applications, by showing that these systems only release the loaded cargo upon the application of an alternating magnetic field, that causes a local heating effect (hyperthermia), also synergistically inducing cell death.

## 4. Conclusions

In this work, both aqueous and solid magnetoliposomes containing calcium ferrite nanoparticles were developed. The nanosystems obtained, with sizes below or around 170 nm, revealed to be promising as nanocarriers for two new thienopyridine-based anticancer drugs active against breast cancer (including the highly aggressive MDA-MB-231 cells). Solid and aqueous magnetoliposomes were able to interact with model membranes (GUVs) by fusion. Considering the high drug encapsulation efficiencies, the lack of toxicity of the drug-loaded systems and the selective antitumor activity of both compounds, the drug-loaded magnetoliposomes containing CaFe_2_O_4_ nanoparticles are promising to future applications in dual therapy (combining magnetic-triggered chemotherapy and magnetic hyperthermia) for breast cancer.

## Figures and Tables

**Table 1:** Growth inhibitory concentrations (GI_50_) of compounds **1** and **2** in breast cancer cell lines.

GI_50_ (μM) [24]	Compound 1	Compound 2
MCF-7	1.2 ± 0.2	1.6 ± 0.3
MDA-MB-231	5.0 ± 0.7	7.0 ± 0.8

**Table 2:** Growth inhibitory concentrations (GI_50_) of compounds **1** and **2** in tumor cell lines and normal (non-tumor) PLP2 cells.

GI_50_ (μM) ^a^	Compound 1	Compound 2
NCI-H460	34.6 ± 1.0	30.4 ± 1.3
HeLa	13.7 ± 0.6	13.0 ± 0.7
HepG2	25.4 ± 2.5	24.2 ± 1.5
PLP2	34.2 ± 3.3	46.8 ± 2.1

^a^ GI50 values correspond to the concentration which inhibited 50% of cell growth. Results are from three independent experiments (performed in triplicate), and are expressed as mean ± standard deviation (SD).

**Table 3:** Selected Rietveld analysis parameters.

Sample	O*_x,y,z_*	*i* *	Lattice Constant (nm)	Size (nm)	R_F_	χ^2^
CaFe_2_O_4_ (original XRD data)	0.243	0.85	0.8340	4.83	2.90	1.06
CaFe_2_O_4_ (background subtracted XRD data)	0.241	0.85	0.8338	5.26	4.98	0.91

* fixed value.

**Table 4:** Size and polydispersity of solid and aqueous magnetoliposomes determined by dynamic light scattering (DLS) (SD: standard deviation).

System	Lipid	Size ± SD (nm)	PDI ± SD
AMLs	Egg-PC	129 ± 12	0.139 ± 0.04
	DPPC	132 ± 15	0.185 ± 0.08
SMLs	DPPC	164 ± 23	0.203 ± 0.02

**Table 5:** Steady-state fluorescence anisotropy (*r*) values for the antitumor compounds **1** and **2** in magnetoliposomes and comparison with the values in neat liposomes.

	Lipid	Anisotropy, r	
		Compound 1	Compound 2
Liposomes	Egg-PC (25 °C)	0.182 [39]	0.155
	DPPC (25 °C)	0.251 [39]	0.181
	DPPC (55 °C)	0.157 [39]	0.115
AMLs	Egg-PC (25 °C)	0.176	0.138
	DPPC (25 °C)	0.232	0.189
	DPPC (55 °C)	0.106	0.105
SMLs	DPPC (25 °C)	0.212	0.175
	DPPC (55 °C)	0.094	0.106
Glycerol	----	0.308 [39]	0.255

**Table 6:** Encapsulation efficiencies of antitumor compounds **1** and **2** in both AMLs and SMLs.

System	*EE%* ± SD	
	Compound 1	Compound 2
AMLs (Egg-PC)	81.9 ± 8.5	86.3 ± 8.2
SMLs (DPPC)	68.2 ± 9.1	75.8 ± 10.9
